# Supplementary material for: Assessing Smoking Status and Risk of SARS-CoV-2 Infection: A Machine Learning Approach among Veterans
Source: Healthcare (Basel). 2022 Jul 4;10(7):1244. doi: 10.3390/healthcare10071244 (PMC9319659; doi:10.3390/healthcare10071244)
Supplement: Supplementary file 1 [file healthcare-10-01244-s001.zip › healthcare-1733406-supplementary.pdf]

healthcare

MDPI

Article

# Title: Assessing Smoking Status and Risk of SARS CoV-2 Infection: A Machine Learning Approach among Veterans

**Alice B. S. Nono Djotsa [1,2,3], Drew A. Helmer [1,2], Catherine Park [1,2,3], Kristine E. Lynch [4], Amir Sharafkhaneh [1,2], Aanand D. Naik [1,5], Javad Razjouyan [1,2,*], Christopher I. Amos [2]**

1 VA HSR&D Center for Innovations in Quality, Effectiveness and Safety, Michael E. DeBakey VA Medical Center, Houston, TX 77030,USA; Alice.NonoDjotsa@va.gov (A.N.D), Drew.Helmer@va.gov (D.H.), catherine.park@va.gov (C.P.), SHARAFKHANEH.AMIR@va.gov (A.S.), Aanand.Naik@va.gov (A.N.), javad.razjouyan@va.gov (J.R.).

2 Department of Medicine, Baylor College of Medicine, Houston, TX 77030, USA; Alice.NonoDjotsa@bcm.edu (A.N.D.), Drew.Helmer@bcm.edu (D.H.), catherine.park@bcm.edu (C.P.), amirs@bcm.edu (A.S.), javad.razjouyan@bcm.edu (JR), Chris.Amos@bcm.edu (C.A.).

3 Big Data Scientist Training Enhancement Program (BD-STEP), VA Office of Research and Development, Washington, DC.

4 VA Salt Lake City Health Care System and University of Utah, Salt Lake City, UT, 84148 USA; Kristine.Lynch@va.gov (K.L.)

5 Department of Management, Policy and Community Health, UTHealth School of Public Health, Houston, TX 77030, USA; aanand.naik@uth.tmc.edu (A.N.)

* Correspondence: javad.razjouyan@bcm.edu ; javad.razjouyan@va.gov; Tel.: +1-713-798-7928

**Citation:** Nono Djotsa, A. B. S.; Helmer, A. D.; Park, C.; Lynch, E. K.; Sharafkhaneh, A.; Naik, D. A.; Razjouyan, J.; Amos, I. C. Assessing Smoking Status and Risk of SARS-CoV-2 Infection: A Machine Learning Approach among Veterans. *Healthcare* **2022**, *10*, 1244. https://doi.org/10.3390/healthcare10071244

Academic Editor: Marco Dettori

Received: 3 May 2022
Accepted: 27 June 2022
Published: 4 July 2022

**Publisher's Note:** MDPI stays neutral with regard to jurisdictional claims in published maps and institutional affiliations.

**Copyright:** © 2022 by the authors. Submitted for possible open access publication under the terms and conditions of the Creative Commons Attribution (CC BY) license (https://creativecommons.org/licenses/by/4.0/).

## Supplementary Materials

**Table S1**. Full list of all patients' characteristics.

| Variables | Never Smoker n= 380648 (32.3%) | Former Smoker n=456348 (38.8%) | p-value | Current Smoker n= 221515 (18.8%) | p-value | Unknown n = 117795 (10.0%) | p-value |
|---|---|---|---|---|---|---|---|
| Tested positive | 85605 (22.5) | 93665 (20.5) | 0.0001 | 26809 (12.1) | 0.0001 | 24171 (20.5) | 0.0001 |
| Sex (Male) | 318343 (83.6) | 422136 (92.5) | 0.0001 | 202063 (91.2) | 0.0001 | 74857 (63.5) | 0.0001 |
| *Race* | | | | | | | |
| White | 239184 (62.8) | 332558 (72.9) | 0.0001 | 150741 (68.1) | 0.0001 | 47987 (40.7) | 0.0001 |
| Black | 101211 (26.6) | 83695 (18.3) | 0.0001 | 53037 (23.9) | 0.0001 | 17251 (14.6) | 0.0001 |
| Others | 40253 (10.6) | 40095 (8.8) | 0.0001 | 17737 (8.0) | 0.0001 | 52557 (44.6) | 0.0001 |
| *Ethnicity* | | | | | | | |
| Non-Hispanic or Latino | 325720 (85.6) | 406810 (89.1) | 0.0001 | 202003 (91.2) | 0.0001 | 64420 (54.7) | 0.0001 |
| Hispanic Or Latino | 41593 (10.9) | 34679 (7.6) | 0.0001 | 12506 (5.6) | 0.0001 | 7567 (6.4) | 0.0001 |
| Unknown | 13335 (3.5) | 14859 (3.3) | 0.0001 | 7006 (3.2) | 0.0001 | 45808 (38.9) | 0.0001 |
| **Age [Mean, SD]** | | | | | | | |
| Age 18–30 | 17299 (4.5) | 10373 (2.3) | 0.0001 | 9122 (4.1) | 0.0001 | 15584 (13.2) | 0.0001 |
| Age 30–40 | 42223 (11.1) | 34708 (7.6) | 0.0001 | 26861 (12.1) | 0.0001 | 26233 (22.3) | 0.0001 |
| Age 40–50 | 50187 (13.2) | 35889 (7.9) | 0.0001 | 23508 (10.6) | 0.0001 | 20558 (17.5) | 0.0001 |
| Age 50–65 | 116777 (30.7) | 111103 (24.3) | 0.0001 | 83803 (37.8) | 0.0001 | 28990 (24.6) | 0.0001 |
| Age 65–75 | 101991 (26.8) | 172289 (37.8) | 0.0001 | 64754 (29.2) | 0.0001 | 15546 (13.2) | 0.0001 |
| Age 75–85 | 36647 (9.6) | 67834 (14.9) | 0.0001 | 11668 (5.3) | 0.0001 | 6539 (5.6) | 0.0001 |
| Age > 85 | 15524 (4.1) | 24152 (5.3) | 0.0001 | 1799 (0.8) | 0.0001 | 4344 (3.7) | 0.0001 |
| ***BMI* [Mean, SD]** | | | | | | | |
| BMI < 18.5 | 3048 (0.8) | 5820 (1.3) | 0.0001 | 6732 (3.0) | 0.0001 | 1919 (1.6) | 0.0001 |
| 18.5 ≤ BMI <30 | 188058 (49.4) | 236762 (51.9) | 0.0001 | 133593 (60.3) | 0.0001 | 70140 (59.5) | 0.0001 |
| BMI ≥ 30 | 189542 (49.8) | 213766 (46.8) | 0.0001 | 81190 (36.7) | 0.0001 | 45736 (38.8) | 0.0001 |
| **Pre-existing Conditions** | | | | | | | |
| Acute Cardiac Injury, N(%) | 6715 (1.8) | 13242 (2.9) | 0.0001 | 5647 (2.5) | 0.0001 | 1144 (1.0) | 0.0001 |
| Acute Liver Injury, N(%) | 551 (0.1) | 943 (0.2) | 0.0001 | 653 (0.3) | 0.0001 | 146 (0.1) | > 0.05 |
| Acute Myocardial Infarction, N(%) | 6703 (1.8) | 13210 (2.9) | 0.0001 | 5637 (2.5) | 0.0001 | 1142 (1.0) | 0.0001 |
| Acute Respiratory Failure, N(%) | 12331 (3.2) | 29309 (6.4) | 0.0001 | 12831 (5.8) | 0.0001 | 2591 (2.2) | 0.0001 |
| Acute Rheumatic Heart Disease, N(%) | 19 (0.0) | 29 (0.0) | | 11 (0.0) | | 3 (0.0) | |
| Acute Kidney Failure, N(%) | 22825 (6.0) | 40092 (8.8) | 0.0001 | 16371 (7.4) | 0.0001 | 4066 (3.5) | 0.0001 |
| Alcohol Dependence, N(%) | 63960 (16.8) | 102131 (22.4) | 0.0001 | 85961 (38.8) | 0.0001 | 7732 (6.6) | 0.0001 |
| Anxiety, N(%) | 89184 (23.4) | 99137 (21.7) | 0.0001 | 60585 (27.4) | 0.0001 | 9058 (7.7) | 0.0001 |
| Acute Respiratory Distress Syndrome, N(%) | 206 (0.1) | 373 (0.1) | > 0.05 | 183 (0.1) | > 0.05 | 38 (0.0) | 0.0001 |
| Asthma, N(%) | 32606 (8.6) | 32184 (7.1) | 0.0001 | 11261 (5.1) | 0.0001 | 2572 (2.2) | 0.0001 |
| Bipolar Disorder, N(%) | 14122 (3.7) | 19188 (4.2) | 0.0001 | 17678 (8.0) | 0.0001 | 2074 (1.8) | 0.0001 |

| | | | | | | | |
|---|---|---|---|---|---|---|---|
| Breastfeeding, N(%) | 475 (0.1) | 236 (0.1) | > 0.05 | 88 (0.0) | 0.0001 | 80 (0.1) | > 0.05 |
| Breastfeed At Index | 10 (0.0) | 4 (0.0) | | 3 (0.0) | | 1 (0.0) | |
| Bronchitis, N(%) | 25599 (6.7) | 36357 (8.0) | 0.0001 | 19134 (8.6) | 0.0001 | 2687 (2.3) | 0.0001 |
| Coronary Atherosclerosis and other Heart Disease, N(%) | 63009 (16.6) | 118778 (26.0) | 0.0001 | 42141 (19.0) | 0.0001 | 7274 (6.2) | 0.0001 |
| Cancer, N(%) | 73409 (19.3) | 116775 (25.6) | 0.0001 | 48138 (21.7) | 0.0001 | 8330 (7.1) | 0.0001 |
| Cardiomyopathy, N(%) | 11670 (3.1) | 20183 (4.4) | 0.0001 | 7878 (3.6) | 0.0001 | 1388 (1.2) | 0.0001 |
| Cerebrovascular Disease, N(%) | 6863 (1.8) | 11416 (2.5) | 0.0001 | 4570 (2.1) | 0.0001 | 1044 (0.9) | 0.0001 |
| Congestive Heart Failure, N(%) | 24805 (6.5) | 47469 (10.4) | 0.0001 | 15537 (7.0) | 0.0001 | 3671 (3.1) | 0.0001 |
| Chronic hepatitis, N(%) | 779 (0.2) | 1436 (0.3) | 0.0001 | 1351 (0.6) | 0.0001 | 169 (0.1) | 0.0001 |
| Chronic Lung Disease, N(%) | 100324 (26.4) | 171849 (37.7) | 0.0001 | 92835 (41.9) | 0.0001 | 11099 (9.4) | 0.0001 |
| Chronic Neurological Disease, N(%) | 14728 (3.9) | 17366 (3.8) | 0.018 | 5928 (2.7) | 0.0001 | 2352 (2.0) | 0.0001 |
| Chronic Rheumatic Heart Disease, N(%) | 3376 (0.9) | 5638 (1.2) | 0.0001 | 1644 (0.7) | 0.0001 | 420 (0.4) | 0.0001 |
| Cirrhosis, N(%) | 6895 (1.8) | 13333 (2.9) | 0.0001 | 8418 (3.8) | 0.0001 | 1659 (1.4) | 0.0001 |
| Chronic Kidney Disease, N(%) | 49213 (12.9) | 76070 (16.7) | 0.0001 | 22267 (10.1) | 0.0001 | 5697 (4.8) | 0.0001 |
| Chronic Kidney Failure, N(%) | 5853 (1.5) | 8660 (1.9) | 0.0001 | 2485 (1.1) | 0.0001 | 997 (0.8) | 0.0001 |
| Complex Sleep Apnea, N(%) | 72 (0.0) | 82 (0.0) | | 29 (0.0) | | 8 (0.0) | |
| Chronic Obstructive Pulmonary Disease, N(%) | 36137 (9.5) | 101866 (22.3) | 0.0001 | 64124 (28.9) | 0.0001 | 5982 (5.1) | 0.0001 |
| Central sleep Apnea Primary, N(%) | 1472 (0.4) | 1951 (0.4) | > 0.05 | 649 (0.3) | 0.0001 | 128 (0.1) | 0.0001 |
| Central serous retinopathy, N(%) | 811 (0.2) | 1295 (0.3) | 0.0001 | 379 (0.2) | > 0.05 | 72 (0.1) | 0.0001 |
| Cardiovascular Disease, N(%) | 113136 (29.7) | 189991 (41.6) | 0.0001 | 74726 (33.7) | 0.0001 | 13663 (11.6) | 0.0001 |
| Dementia, N(%) | 14937 (3.9) | 23270 (5.1) | 0.0001 | 6417 (2.9) | 0.0001 | 4435 (3.8) | 0.123 |
| Diabetes Any, N(%) | 119904 (31.5) | 170917 (37.5) | 0.0001 | 61119 (27.6) | 0.0001 | 12753 (10.8) | 0.0001 |
| Diabetes Other, N(%) | 11692 (3.1) | 17364 (3.8) | 0.0001 | 6193 (2.8) | 0.0001 | 1226 (1.0) | 0.0001 |
| Diabetes Type 1, N(%) | 6767 (1.8) | 9445 (2.1) | 0.0001 | 3445 (1.6) | 0.0001 | 728 (0.6) | 0.0001 |
| Diabetes Type 2, N(%) | 118959 (31.3) | 169769 (37.2) | 0.0001 | 60575 (27.3) | 0.0001 | 12548 (10.7) | 0.0001 |
| Diabetes With Complications, N(%) | 83422 (21.9) | 123064 (27.0) | 0.0001 | 42028 (19.0) | 0.0001 | 8772 (7.4) | 0.0001 |
| Diabetes Without Complications, N(%) | 108246 (28.4) | 154275 (33.8) | 0.0001 | 54757 (24.7) | 0.0001 | 10576 (9.0) | 0.0001 |
| Drug Dependence, N(%) | 10998 (2.9) | 23280 (5.1) | 0.0001 | 34315 (15.5) | 0.0001 | 2826 (2.4) | 0.0001 |
| Emphysema, N(%) | 3092 (0.8) | 12544 (2.7) | 0.0001 | 8342 (3.8) | 0.0001 | 597 (0.5) | 0.0001 |
| Guillain-Barre Syndrome, N(%) | 235 (0.1) | 306 (0.1) | > 0.05 | 107 (0.0) | 0.0001 | 20 (0.0) | 0.0001 |
| Heart Disease, N(%) | 81153 (21.3) | 145589 (31.9) | 0.0001 | 52375 (23.6) | 0.0001 | 9872 (8.4) | 0.0001 |
| Heart Failure, N(%) | 31152 (8.2) | 58606 (12.8) | 0.0001 | 19333 (8.7) | 0.0001 | 4552 (3.9) | 0.0001 |
| Hemorrhagic Stroke, N(%) | 925 (0.2) | 1379 (0.3) | 0.0001 | 531 (0.2) | > 0.05 | 170 (0.1) | 0.0001 |
| Human Immunodeficiency Virus, N(%) | 3354 (0.9) | 3766 (0.8) | 0.0001 | 2736 (1.2) | 0.0001 | 828 (0.7) | 0.0001 |
| Hypertension, N(%) | 226370 (59.5) | 316762 (69.4) | 0.0001 | 132837 (60.0) | 0.00013 | 22791 (19.3) | 0.0001 |
| Hyperlipidemia, N(%) | 218820 (57.5) | 297932 (65.3) | 0.0001 | 121331 (54.8) | 0.0001 | 17705 (15.0) | 0.0001 |
| Inflammatory bowel Disease, N(%) | 12803 (3.4) | 17101 (3.7) | 0.0001 | 7833 (3.5) | 0.0404 | 1622 (1.4) | 0.0001 |
| Influenza, N(%) | 7202 (1.9) | 8856 (1.9) | > 0.05 | 4650 (2.1) | 0.0001 | 1182 (1.0) | 0.0001 |
| Ischemic Stroke, N(%) | 15716 (4.1) | 25963 (5.7) | 0.0001 | 10702 (4.8) | 0.0001 | 2479 (2.1) | 0.0001 |
| Kidney Disease All, N(%) | 82051 (21.6) | 121420 (26.6) | 0.0001 | 43206 (19.5) | 0.0001 | 9712 (8.2) | 0.0001 |

| | | | | | | | |
|---|---|---|---|---|---|---|---|
| Liver Disease, N(%) | 25722 (6.8) | 36334 (8.0) | 0.0001 | 19167 (8.7) | 0.0001 | 3199 (2.7) | 0.0001 |
| Lower Respiratory Infection, N(%) | 32746 (8.6) | 38228 (8.4) | 0.0011 | 18067 (8.2) | 0.0001 | 4171 (3.5) | 0.0001 |
| Major Depressive Disorder, N(%) | 138385 (36.4) | 157528 (34.5) | 0.0001 | 96981 (43.8) | 0.0001 | 14395 (12.2) | 0.0001 |
| Measles, N(%) | 10 (0.0) | 9 (0.0) | | 4 (0.0) | | 3 (0.0) | |
| Multiple Sclerosis, N(%) | 1757 (0.5) | 2051 (0.4) | 0.0001 | 852 (0.4) | 0.0001 | 349 (0.3) | 0.0001 |
| Nephrosis, N(%) | 10643 (2.8) | 16172 (3.5) | 0.0001 | 4765 (2.2) | 0.0001 | 1181 (1.0) | 0.0001 |
| Obesity Hypoventilation, N(%) | 1579 (0.4) | 2519 (0.6) | 0.0001 | 759 (0.3) | 0.0001 | 230 (0.2) | 0.0001 |
| Obstructive Sleep Apnea, N(%) | 128680 (33.8) | 152873 (33.5) | 0.0001 | 55524 (25.1) | 0.0001 | 9929 (8.4) | 0.0001 |
| Other and Ill-Defined Heart Disease, N(%) | 10359 (2.7) | 16653 (3.6) | 0.0001 | 5996 (2.7) | > 0.05 | 1392 (1.2) | 0.0001 |
| Overweight, N(%) | 29827 (7.8) | 31900 (7.0) | 0.0001 | 10319 (4.7) | 0.0001 | 4907 (4.2) | 0.0001 |
| Peripheral Artery Disease, N(%) | 34538 (9.1) | 67723 (14.8) | 0.0001 | 31710 (14.3) | 0.0001 | 5122 (4.3) | 0.0001 |
| Parkinsons Disease, N(%) | 5125 (1.3) | 7112 (1.6) | 0.0001 | 1425 (0.6) | 0.0001 | 951 (0.8) | 0.0001 |
| Polycystic Ovary Syndrome, N(%) | 1067 (0.3) | 482 (0.1) | 0.0001 | 241 (0.1) | 0.0001 | 62 (0.1) | 0.0001 |
| Pneumonia, N(%) | 15814 (4.2) | 31011 (6.8) | 0.0001 | 13667 (6.2) | 0.0001 | 3082 (2.6) | 0.0001 |
| Ventilator-Associated Pneumonia, N(%) | 64 (0.0) | 129 (0.0) | | 64 (0.0) | | 30 (0.0) | |
| Pregnancy, N(%) | 2097 (0.6) | 1200 (0.3) | 0.0001 | 543 (0.2) | 0.0001 | 583 (0.5) | 0.0001 |
| Pregnancy at Index, N(%) | 490 (0.1) | 324 (0.1) | > 0.05 | 146 (0.1) | > 0.05 | 234 (0.2) | 0.0001 |
| Posttraumatic Stress Disorder, N(%) | 100159 (26.3) | 115852 (25.4) | 0.0001 | 68945 (31.1) | 0.0001 | 10316 (8.8) | 0.0001 |
| Pulmonary Heart Disease, N(%) | 6676 (1.8) | 13010 (2.9) | 0.0001 | 4050 (1.8) | > 0.05 | 959 (0.8) | 0.0001 |
| Schizophrenia, N(%) | 7612 (2.0) | 10818 (2.4) | 0.0001 | 11573 (5.2) | 0.0001 | 1859 (1.6) | 0.0001 |
| Septic Shock, N(%) | 7577 (2.0) | 14012 (3.1) | 0.0001 | 5810 (2.6) | 0.0001 | 2015 (1.7) | 0.0001 |
| Sickle Cell Disease, N(%) | 868 (0.2) | 637 (0.1) | 0.0001 | 344 (0.2) | > 0.05 | 67 (0.1) | 0.0001 |
| Sleep Related Non-Obstructive Hypoventilation, N(%) | 1300 (0.3) | 2490 (0.5) | 0.0001 | 849 (0.4) | 0.0001 | 124 (0.1) | 0.0001 |
| Spleen Removed, N(%) | 517 (0.1) | 759 (0.2) | 0.0001 | 380 (0.2) | 0.0001 | 68 (0.1) | > 0.05 |
| Urinary Stones, N(%) | 38176 (10.0) | 52765 (11.6) | 0.0001 | 19089 (8.6) | 0.0001 | 4276 (3.6) | 0.0001 |
| Venous Thromboembolism, N(%) | 9158 (2.4) | 13571 (3.0) | 0.0001 | 5474 (2.5) | 0.0159 | 1232 (1.0) | 0.0001 |
| **Pre-infection Medications** | | | | | | | |
| Acetyl Cholinesterase (ACE) Inhibitor, N(%) | 99074 (26.0) | 144906 (31.8) | 0.0001 | 65228 (29.4) | 0.0001 | 9944 (8.4) | 0.0001 |
| Acetaminophen, N(%) | 140220 (36.8) | 187679 (41.1) | 0.0001 | 97082 (43.8) | 0.0001 | 18717 (15.9) | 0.0001 |
| Androgen deprivation therapy, N(%) | 4172 (1.1) | 6012 (1.3) | 0.0001 | 2102 (0.9) | 0.0001 | 470 (0.4) | 0.0001 |
| Albumin, N(%) | 1896 (0.5) | 3682 (0.8) | 0.0001 | 1589 (0.7) | 0.0001 | 456 (0.4) | 0.0001 |
| Allopurinol, N(%) | 24223 (6.4) | 33987 (7.4) | 0.0001 | 8989 (4.1) | 0.0001 | 1947 (1.7) | 0.0001 |
| Alphablocker, N(%) | 99655 (26.2) | 149798 (32.8) | 0.0001 | 61513 (27.8) | 0.0001 | 10161 (8.6) | 0.0001 |
| Antianginal, N(%) | 25447 (6.7) | 48572 (10.6) | 0.0001 | 17027 (7.7) | 0.0001 | 2818 (2.4) | 0.0001 |
| Antiarrhythmic, N(%) | 7916 (2.1) | 14177 (3.1) | 0.0001 | 3901 (1.8) | 0.0001 | 976 (0.8) | 0.0001 |
| Antibiotic, N(%) | 156249 (41.0) | 205848 (45.1) | 0.0001 | 101168 (45.7) | 0.0001 | 20365 (17.3) | 0.0001 |
| Anticoagulant Inpatient, N(%) | 43903 (11.5) | 79260 (17.4) | 0.0001 | 36785 (16.6) | 0.0001 | 7715 (6.5) | 0.0001 |
| Anticoagulant Outpatient, N(%) | 51501 (13.5) | 91730 (20.1) | 0.0001 | 31645 (14.3) | 0.0001 | 5594 (4.7) | 0.0001 |
| Antihistamine, N(%) | 163405 (42.9) | 195617 (42.9) | > 0.05 | 98457 (44.4) | 0.0001 | 16242 (13.8) | 0.0001 |

| | | | | | | | |
|---|---|---|---|---|---|---|---|
| Antipsychotic, N(%) | 30686 (8.1) | 41224 (9.0) | 0.0001 | 34934 (15.8) | 0.0001 | 4585 (3.9) | 0.0001 |
| Antiviral, N(%) | 34109 (9.0) | 37739 (8.3) | 0.0001 | 19329 (8.7) | 0.0001 | 5948 (5.0) | 0.0001 |
| Angiotensin II Receptor Blocker, N(%) | 51761 (13.6) | 71243 (15.6) | 0.0001 | 22206 (10.0) | 0.0001 | 4153 (3.5) | 0.0001 |
| Aromatase Inhibitors, N(%) | 825 (0.2) | 525 (0.1) | 0.0001 | 246 (0.1) | 0.0001 | 58 (0.0) | 0.0001 |
| Azithromycin, N(%) | 35610 (9.4) | 51654 (11.3) | 0.0001 | 26702 (12.1) | 0.0001 | 4594 (3.9) | 0.0001 |
| Bacillus Calmette-Guerin, N(%) | 404 (0.1) | 1188 (0.3) | 0.0001 | 435 (0.2) | 0.0001 | 51 (0.0) | 0.0001 |
| Benzodiazepine, N(%) | 42551 (11.2) | 59332 (13.0) | 0.0001 | 36452 (16.5) | 0.0001 | 5207 (4.4) | 0.0001 |
| Beta Agonist Nebulizer, N(%) | 26255 (6.9) | 53791 (11.8) | 0.0001 | 28165 (12.7) | 0.0001 | 4485 (3.8) | 0.0001 |
| Beta Blocker, N(%) | 112848 (29.6) | 177470 (38.9) | 0.0001 | 71001 (32.1) | 0.0001 | 12367 (10.5) | 0.0001 |
| Bronchodilator, N(%) | 75167 (19.7) | 130087 (28.5) | 0.0001 | 74386 (33.6) | 0.0001 | 8600 (7.3) | 0.0001 |
| Calcium Channel Blocker, N(%) | 93177 (24.5) | 132587 (29.1) | 0.0001 | 57700 (26.0) | 0.0001 | 9387 (8.0) | 0.0001 |
| Chloroquine, N(%) | 9 (0.0) | 9 (0.0) | | 2 (0.0) | | 0 (0.0) | |
| Cis-atracurium, N(%) | 64 (0.0) | 114 (0.0) | | 63 (0.0) | | 10 (0.0) | |
| Colchicine, N(%) | 9877 (2.6) | 13471 (3.0) | 0.0001 | 4338 (2.0) | 0.0001 | 850 (0.7) | 0.0001 |
| Corticosteroid, N(%) | 138794 (36.5) | 189501 (41.5) | 0.0001 | 86454 (39.0) | 0.0001 | 13861 (11.8) | 0.0001 |
| Degarelix, N(%)yrs | 71 (0.0) | 89 (0.0) | | 34 (0.0) | | 15 (0.0) | |
| Dexamethasone, N(%) | 13688 (3.6) | 18955 (4.2) | 0.0001 | 8981 (4.1) | 0.0001 | 1835 (1.6) | 0.0001 |
| Dobutamine, N(%) | 341 (0.1) | 709 (0.2) | 0.0001 | 213 (0.1) | > 0.05 | 43 (0.0) | 0.0001 |
| Eculizumab, N(%) | 7 (0.0) | 8 (0.0) | | 3 (0.0) | | 0 (0.0) | |
| Epinephrine, N(%) | 11341 (3.0) | 12637 (2.8) | 0.0001 | 5657 (2.6) | 0.0001 | 1060 (0.9) | 0.0001 |
| Estrogen, N(%) | 12269 (3.2) | 7536 (1.7) | 0.0001 | 3680 (1.7) | 0.0001 | 911 (0.8) | 0.0001 |
| Famotidine, N(%) | 32110 (8.4) | 42797 (9.4) | 0.0001 | 19202 (8.7) | 0.0001 | 3316 (2.8) | 0.0001 |
| Furosemide, N(%) | 37342 (9.8) | 68350 (15.0) | 0.0001 | 22713 (10.3) | 0.0001 | 5194 (4.4) | 0.0001 |
| Glucocorticoid, N(%) | 83724 (22.0) | 112888 (24.7) | 0.0001 | 56713 (25.6) | 0.0001 | 9589 (8.1) | 0.0001 |
| Histamine 2 Receptor Blocker, N(%) | 33603 (8.8) | 43993 (9.6) | 0.0001 | 19824 (8.9) | > 0.05 | 3187 (2.7) | 0.0001 |
| Hydrochlorothiazide, N(%) | 60649 (15.9) | 77779 (17.0) | 0.0001 | 33962 (15.3) | 0.0001 | 5139 (4.4) | 0.0001 |
| Hydroxychloroquine, N(%) | 3291 (0.9) | 3989 (0.9) | > 0.05 | 1382 (0.6) | 0.0001 | 264 (0.2) | 0.0001 |
| Inhaled Corticosteroids, N(%) | 7947 (2.1) | 11208 (2.5) | 0.0001 | 4006 (1.8) | 0.0001 | 679 (0.6) | 0.0001 |
| Inhaled Corticosteroids and Long-Acting Beta-Agonist, N(%) | 29494 (7.7) | 61378 (13.4) | 0.0001 | 33023 (14.9) | 0.0001 | 3170 (2.7) | 0.0001 |
| Induction Ventilation Medications, N(%) | 6615 (1.7) | 10240 (2.2) | 0.0001 | 4827 (2.2) | 0.0001 | 935 (0.8) | 0.0001 |
| Inhaled Budesonide, N(%) | 1290 (0.3) | 1999 (0.4) | 0.0001 | 575 (0.3) | > 0.05 | 156 (0.1) | 0.0001 |
| Inhaled Epo-prostenol, N(%) | 29 (0.0) | 69 (0.0) | | 16 (0.0) | | 6 (0.0) | |
| Isoproterenol, N(%) | 35 (0.0) | 66 (0.0) | | 24 (0.0) | | 7 (0.0) | |
| Long-Acting Beta-Agonist, N(%) | 766 (0.2) | 2402 (0.5) | 0.0001 | 974 (0.4) | 0.0001 | 106 (0.1) | 0.0001 |
| Lopinavir, N(%) | 20 (0.0) | 30 (0.0) | | 24 (0.0) | | 5 (0.0) | |
| Losartan, N(%) | 47113 (12.4) | 63735 (14.0) | 0.0001 | 20044 (9.0) | 0.0001 | 3754 (3.2) | 0.0001 |
| Metformin, N(%) | 72749 (19.1) | 100442 (22.0) | 0.0001 | 38267 (17.3) | 0.0001 | 6021 (5.1) | 0.0001 |
| Milrinone, N(%) | 181 (0.0) | 370 (0.1) | 0.0001 | 101 (0.0) | | 19 (0.0) | |
| Norepinephrine, N(%) | 1289 (0.3) | 2775 (0.6) | 0.0001 | 1097 (0.5) | 0.0001 | 330 (0.3) | 0.975 |
| Non-steroidal Anti-Inflammatory Drug, N(%) | 235755 (61.9) | 285628 (62.6) | 0.0001 | 145315 (65.6) | 0.0001 | 27160 (23.1) | 0.0001 |

| | | | | | | | |
|---|---|---|---|---|---|---|---|
| Opioid, N(%) | 16907 (4.4) | 28996 (6.4) | 0.0001 | 17443 (7.9) | 0.0001 | 2676 (2.3) | 0.0001 |
| Platelet Aggregation Inhibitor, N(%) | 20287 (5.3) | 40000 (8.8) | 0.0001 | 16090 (7.3) | 0.0001 | 2466 (2.1) | 0.0001 |
| Phenylephrine, N(%) | 6486 (1.7) | 9446 (2.1) | 0.0001 | 4064 (1.8) | 0.004 | 753 (0.6) | 0.0001 |
| Proton Pump Inhibitor, N(%) | 135932 (35.7) | 191245 (41.9) | 0.0001 | 84305 (38.1) | 0.0001 | 13200 (11.2) | 0.0001 |
| Remdesivir, N(%) | 10 (0.0) | 32 (0.0) | | 4 (0.0) | | 2 (0.0) | |
| Ribavirin, N(%) | 25 (0.0) | 54 (0.0) | | 62 (0.0) | | 8 (0.0) | |
| Sarilumab, N(%) | 1 (0.0) | 9 (0.0) | | 2 (0.0) | | 0 (0.0) | |
| Selective Estrogen Receptor Degrader, N(%) | 19 (0.0) | 18 (0.0) | | 9 (0.0) | | 4 (0.0) | |
| Selective Estrogen Receptor Modulators, N(%) | 469 (0.1) | 355 (0.1) | > 0.05 | 107 (0.0) | 0.0001 | 30 (0.0) | 0.0001 |
| Serotonin and Norepinephrine Reuptake Inhibitors, N(%) | 52973 (13.9) | 68321 (15.0) | 0.0001 | 36673 (16.6) | 0.0001 | 4533 (3.8) | 0.0001 |
| Selective Serotonin Reuptake Inhibitors, N(%) | 79424 (20.9) | 95112 (20.8) | > 0.05 | 53298 (24.1) | 0.0001 | 8411 (7.1) | 0.0001 |
| Statin, N(%) | 179529 (47.2) | 264318 (57.9) | 0.0001 | 109726 (49.5) | 0.0001 | 16040 (13.6) | 0.0001 |
| Tocilizumab, N(%) | 119 (0.0) | 222 (0.0) | | 75 (0.0) | | 10 (0.0) | |
| Tricyclic, N(%) | 19769 (5.2) | 21785 (4.8) | 0.0001 | 12138 (5.5) | 0.0001 | 1575 (1.3) | 0.0001 |
| Valsartan, N(%) | 5540 (1.5) | 9045 (2.0) | 0.0001 | 2624 (1.2) | 0.0001 | 486 (0.4) | 0.0001 |
| Vasopressin, N(%) | 465 (0.1) | 990 (0.2) | 0.0001 | 345 (0.2) | 0.0001 | 110 (0.1) | > 0.05 |
| Vasopressor, N(%) | 7571 (2.0) | 11270 (2.5) | 0.0001 | 4536 (2.0) | > 0.05 | 1123 (1.0) | 0.0001 |
| Vecuronium, N(%) | 15 (0.0) | 29 (0.0) | | 17 (0.0) | | 6 (0.0) | |

**Table S2a.** List of variables studied and results of most important variable selection from prevalence, Univariate Analysis, LASSO and sequential forward stepwise selection for Imputed data.

| Variables | Prevalence (%) | Univariate Analysis | | LASSO | Forward Selection | Logistic regression Coefficient |
|---|---|---|---|---|---|---|
| | | Beta | pValue | | | |
| Sex (male) | 86.5 | 0.07 | P < 0.0001 | Yes | Yes | |
| Age | | | | | | |
| Age 18–30 | 3.6 | 0.14 | P < 0.0001 | Yes | No | |
| Age 30–40 | 10.8 | 0.13 | P < 0.0001 | Yes | No | |
| Age 40–50 | 10.6 | 0.17 | P < 0.0001 | Yes | No | |
| Age 50–65 | 28.2 | -0.05 | P < 0.0001 | No | No | |
| Age 65–75 | 30.1 | -0.2 | P < 0.0001 | Yes | No | |
| Age 75–85 | 12.2 | 0.01 | P < 0.0001 | No | No | |
| Age > 85 | 4.5 | 0.3 | P < 0.0001 | Yes | Yes | 0.2830 |
| Race | | | | | | |
| White, N(%) | 65.5 | -0.03 | P = 0.0009 | Yes | No | |
| Black , N(%) | 21.7 | 0 | P > 0.05 | No | No | |
| Other , N(%) | 12.8 | 0.06 | P < 0.0001 | Yes | Yes | |
| Ethnicity | | | | | | |
| Hispanic or Latino, N(%) | 8.1 | | P < 0.0001 | Yes | Yes | 0.1606 |
| BMI | | | | | | |
| BMI < 18.5 | 1.5 | 0.29 | P < 0.0001 | Yes | Yes | -0.3120 |
| 18.5 ≤ BMI <30 | 53.4 | | P < 0.0001 | No | | |
| BMI ≥ 30 | 45.1 | | P < 0.0001 | Yes | Yes | 0.2695 |
| Acetyl Cholinesterase (ACE) Inhibitor, N(%) | 27.1 | -0.06 | P < 0.0001 | Yes | No | |
| Acetaminophen, N(%) | 37.3 | -0.19 | P < 0.0001 | Yes | No | |
| Acute Cardiac Injury, N(%) | 2.3 | -0.04 | P = 0.0002 | No | No | |
| Acute Liver Injury, N(%) | < 1% | | P > 0.05 | No | No | |
| Acute Myocardial Infarction, N(%) | 2.3 | -0.04 | P = 0.0002 | No | No | |
| Acute Respiratory Failure, N(%) | 4.9 | 0.1 | P = 0.003 | Yes | No | |
| Acute Rheumatic Heart Disease, N(%) | < 1% | | P > 0.05 | No | No | |
| Androgen deprivation therapy, N(%) | 1.1 | -0.27 | P < 0.0001 | Yes | No | |
| Acute Kidney Failure, N(%) | 7.1 | 0.02 | P = 0.012 | No | No | |
| Albumin, N(%) | < 1% | | P > 0.05 | No | No | |
| Alcohol Dependence, N(%) | 22.1 | -0.16 | P < 0.0001 | No | No | |
| Allopurinol, N(%) | 5.9 | 0.08 | P < 0.0001 | Yes | No | |
| Alphablocker, N(%) | 27.3 | -0.13 | P < 0.0001 | Yes | No | |
| Antianginal, N(%) | 8.0 | -0.1 | P < 0.0001 | No | No | |
| Antiarrhythmic, N(%) | 2.3 | -0.13 | P < 0.0001 | Yes | No | |
| Antibiotic, N(%) | 41.1 | -0.21 | P < 0.0001 | Yes | No | |

| | | | | | | |
|---|---|---|---|---|---|---|
| Anticoagulant Inpatient, N(%) | 14.3 | -0.14 | P < 0.0001 | Yes | No | |
| Anticoagulant Outpatient, N(%) | 15.3 | -0.09 | P < 0.0001 | No | No | |
| Antihistamine, N(%) | 40.3 | -0.14 | P < 0.0001 | No | No | |
| Antipsychotic, N(%) | 9.5 | -0.23 | P < 0.0001 | Yes | No | |
| Antiviral, N(%) | 8.3 | -0.05 | P < 0.0001 | No | No | |
| Anxiety, N(%) | 21.9 | -0.1 | P < 0.0001 | No | No | |
| Angiotensin II Receptor Blocker, N(%) | 12.7 | 0.03 | P < 0.0001 | No | No | |
| Acute Respiratory Distress Syndrome, N(%) | < 1% | | P > 0.05 | No | No | |
| Aromatase Inhibitors, N(%) | < 1% | | P > 0.05 | No | No | |
| Asthma, N(%) | 6.7 | 0 | P > 0.05 | No | No | |
| Azithromycin, N(%) | 10.1 | -0.02 | P < 0.0001 | Yes | No | |
| Bacillus Calmette-Guerin, N(%) | < 1% | | P > 0.05 | No | No | |
| Benzodiazepine, N(%) | 12.2 | -0.26 | P < 0.0001 | Yes | No | |
| Beta Agonist Nebulizer, N(%) | 9.6 | -0.16 | P < 0.0001 | Yes | No | |
| Beta Blocker, N(%) | 31.8 | -0.12 | P < 0.0001 | Yes | No | |
| Bipolar Disorder, N(%) | 4.5 | -0.31 | P < 0.0001 | Yes | No | |
| Bronchitis, N(%) | 7.1 | -0.04 | P < 0.0001 | Yes | No | |
| Bronchodilator, N(%) | 24.5 | -0.17 | P < 0.0001 | No | No | |
| Coronary Atherosclerosis and other Heart Disease, N(%) | 19.7 | -0.06 | P < 0.0001 | Yes | No | |
| Cancer, N(%) | 21.0 | -0.3 | P < 0.0001 | Yes | Yes | -0.3253 |
| Cardiomyopathy, N(%) | 3.5 | -0.17 | P < 0.0001 | Yes | No | |
| Calcium Channel Blocker, N(%) | 24.9 | -0.08 | P < 0.0001 | No | No | |
| Cerebrovascular Disease, N(%) | 2.0 | -0.02 | P > 0.05 | No | No | |
| Congestive Heart Failure, N(%) | 7.8 | -0.08 | P < 0.0001 | Yes | No | |
| Chloroquine, N(%) | < 1% | | P > 0.05 | No | No | |
| Chronic hepatitis, N(%) | < 1% | | P > 0.05 | No | No | |
| Chronic Lung Disease, N(%) | 32.0 | -0.18 | P < 0.0001 | Yes | No | |
| Chronic Neurological Disease, N(%) | 3.4 | 0.07 | P < 0.0001 | Yes | No | |
| Chronic Rheumatic Heart Disease, N(%) | < 1% | | P > 0.05 | No | No | |
| Cirrhosis, N(%) | 2.6 | -0.37 | P < 0.0001 | Yes | No | |
| Cis-atracurium, N(%) | < 1% | | P > 0.05 | No | No | |
| Chronic Kidney Disease, N(%) | 13.0 | 0.03 | P < 0.0001 | Yes | No | |
| Chronic Kidney Failure, N(%) | 1.5 | 0.06 | 0.0021 | No | No | |
| Colchicine, N(%) | 2.4 | 0 | P > 0.05 | No | No | |
| Complex Sleep Apnea, N(%) | < 1% | | P > 0.05 | No | No | |
| Chronic Obstructive Pulmonary Disease, N(%) | 17.7 | -0.24 | P < 0.0001 | No | No | |
| Corticosteroid, N(%) | 36.4 | -0.14 | P < 0.0001 | Yes | No | |
| Central Sleep Apnea Primary, N(%) | < 1% | | P > 0.05 | No | No | |
| Central Serous Retinopathy, N(%) (CRS) | < 1% | | P > 0.05 | No | No | |
| Cardiovascular Disease, N(%) | 33.3 | -0.12 | P < 0.0001 | Yes | No | |

| | | | | | | |
|---|---|---|---|---|---|---|
| Degarelix, N(%) | < 1% | | P > 0.05 | No | No | |
| Dementia, N(%) | 4.2 | 0.38 | P < 0.0001 | Yes | Yes | 0.3506 |
| Dexamethasone, N(%) | 3.7 | -0.17 | P < 0.0001 | No | No | |
| Diabetes Any, N(%) | 31.0 | 0.11 | P < 0.0001 | Yes | Yes | |
| Diabetes Other, N(%) | 3.1 | -0.03 | P = 0.0029 | Yes | Yes | |
| Diabetes Type 1, N(%) | 1.7 | 0 | P > 0.05 | No | No | |
| Diabetes Type 2, N(%) | 30.8 | 0.11 | P < 0.0001 | Yes | No | |
| Diabetes With Complications, N(%) | 21.9 | 0.09 | P < 0.0001 | No | No | |
| Diabetes Without Complications, N(%) | 27.9 | 0.11 | P < 0.0001 | No | No | |
| Dobutamine, N(%) | < 1% | | P > 0.05 | No | No | |
| Non-Alcohol Drug Dependence, N(%) | 6.1 | -0.49 | P < 0.0001 | Yes | Yes | |
| Eculizumab, N(%) | < 1% | | P > 0.05 | No | No | |
| Emphysema, N(%) | 2.1 | -0.33 | P < 0.0001 | Yes | No | |
| Epinephrine, N(%) | 2.6 | -0.11 | P < 0.0001 | Yes | No | |
| Estrogen, N(%) | 2.1 | -0.09 | P < 0.0001 | Yes | No | |
| Famotidine, N(%) | 8.3 | -0.12 | P < 0.0001 | No | No | |
| Furosemide, N(%) | 11.4 | -0.06 | P < 0.0001 | No | No | |
| Guillain-Barre Syndrome, N(%) | < 1% | | P > 0.05 | No | No | |
| Glucocorticoid, N(%) | 22.4 | -0.13 | P < 0.0001 | No | No | |
| Histamine 2 Receptor Blocker, N(%) | 8.6 | -0.19 | P < 0.0001 | Yes | No | |
| Hydrochlorothiazide, N(%) | 15.1 | -0.03 | P = 0.0024 | No | No | |
| Heart Disease, N(%) | 24.6 | -0.07 | P < 0.0001 | Yes | No | |
| Heart Failure, N(%) | 9.7 | -0.06 | P < 0.0001 | No | No | |
| Hemorrhagic Stroke, N(%) | < 1% | | P > 0.05 | No | No | |
| Human Immunodeficiency Virus, N(%) | < 1% | | P > 0.05 | No | No | |
| Hypertension, N(%) | 59.4 | -0.04 | P = 0.0033 | Yes | No | |
| Hydroxychloroquine, N(%) | < 1% | | P > 0.05 | No | No | |
| Hyperlipidemia, N(%) | 55.7 | 0.03 | P < 0.0001 | Yes | No | |
| Inflammatory bowel Disease, N(%) | 3.3 | -0.16 | P < 0.0001 | Yes | No | |
| Inhaled Corticosteroids, N(%) | 2.0 | -0.12 | P < 0.0001 | Yes | No | |
| Inhaled Corticosteroids and Long-Acting Beta-Agonist, N(%) | 10.8 | -0.15 | P < 0.0001 | Yes | No | |
| Induction Ventilation Medications, N(%) | 1.9 | -0.25 | P < 0.0001 | No | No | |
| Influenza, N(%) | 1.9 | 0.09 | P < 0.0001 | Yes | No | |
| Inhaled Budesonide, N(%) | < 1% | | P > 0.05 | No | No | |
| Inhaled Epo-prostenol, N(%) | < 1% | | P > 0.05 | No | No | |
| Ischemic Stroke, N(%) | 4.7 | -0.01 | P > 0.05 | No | No | |
| Isoproterenol, N(%) | < 1% | | P > 0.05 | No | No | |
| Kidney Disease All, N(%) | 21.8 | -0.03 | P < 0.0001 | Yes | No | |
| Long-Acting Beta-Agonist, N(%) | < 1% | | P > 0.05 | No | No | |
| Liver Disease, N(%) | 7.2 | -0.16 | P < 0.0001 | Yes | No | |
| Lopinavir, N(%) | < 1% | | P > 0.05 | No | No | |

| | | | | | | |
|---|---|---|---|---|---|---|
| Losartan, N(%) | 11.4 | 0.03 | P < 0.0001 | No | No | |
| Lower Respiratory Infection, N(%) | 7.9 | 0.13 | P < 0.0001 | Yes | Yes | 0.0888 |
| Major Depressive Disorder, N(%) | 34.6 | -0.13 | P < 0.0001 | Yes | No | |
| Measles, N(%) | < 1% | | P > 0.05 | No | No | |
| Metformin, N(%) | 18.5 | 0.11 | P < 0.0001 | Yes | No | |
| Milrinone, N(%) | < 1% | | P > 0.05 | No | No | |
| Multiple Sclerosis, N(%) | < 1% | | P > 0.05 | No | No | |
| Nephrosis, N(%) | 2.8 | 0 | P > 0.05 | No | No | |
| Norepinephrine, N(%) | < 1% | | P > 0.05 | No | No | |
| Non-steroidal Anti-Inflammatory Drug, N(%) | 59.0 | -0.15 | P < 0.0001 | Yes | No | |
| Obesity Hypoventilation, N(%) | < 1% | | P > 0.05 | No | No | |
| Opioid, N(%) | 5.6 | -0.3 | P < 0.0001 | Yes | No | |
| Obstructive Sleep Apnea, N(%) | 29.5 | 0.05 | P < 0.0001 | No | No | |
| Other and Ill-Defined Heart Disease, N(%) | 2.9 | -0.07 | P < 0.0001 | No | No | |
| Overweight, N(%) | 6.5 | 0.27 | P < 0.0001 | Yes | No | |
| Peripheral Artery Disease, N(%) | 11.8 | -0.19 | P < 0.0001 | Yes | No | |
| Platelet Aggregation Inhibitor, N(%) | 6.7 | -0.06 | P < 0.0001 | Yes | No | |
| Parkinsons Disease, N(%) | 1.2 | 0.12 | P < 0.0001 | Yes | No | |
| Polycystic Ovary Syndrome, N(%) | < 1% | | P > 0.05 | No | No | |
| Phenylephrine, N(%) | 1.8 | -0.27 | P < 0.0001 | Yes | No | -0.2799 |
| Pneumonia, N(%) | 5.4 | 0.12 | P < 0.0001 | Yes | No | 0.1790 |
| Ventilator-Associated Pneumonia, N(%) | < 1% | | P > 0.05 | No | No | |
| Proton Pump Inhibitor, N(%) | 36.1 | -0.15 | P < 0.0001 | Yes | No | |
| Pregnancy, N(%) | < 1% | | P > 0.05 | No | No | |
| Pregnancy at Index, N(%) | < 1% | | P > 0.05 | No | No | |
| Posttraumatic Stress Disorder, N(%) | 25.1 | -0.1 | P < 0.0001 | No | No | |
| Pulmonary Heart Disease, N(%) | 2.1 | -0.12 | P < 0.0001 | Yes | Yes | |
| Remdesivir, N(%) | < 1% | | P > 0.05 | No | No | |
| Ribavirin, N(%) | < 1% | | P > 0.05 | No | No | |
| Rosiglitazone, N(%) | < 1% | | P > 0.05 | No | No | |
| Sarilumab, N(%) | < 1% | | P > 0.05 | No | No | |
| Schizophrenia, N(%) | 2.7 | -0.23 | P < 0.0001 | Yes | No | |
| Septic Shock, N(%) | 2.5 | 0.12 | P < 0.0001 | Yes | Yes | 0.0675 |
| Selective Estrogen Receptor Degrader, N(%) | < 1% | | P > 0.05 | No | No | |
| Selective Estrogen Receptor Modulators, N(%) | < 1% | | P > 0.05 | No | No | |
| Sickle Cell Disease, N(%) | < 1% | | P > 0.05 | No | No | |
| Sleep Related Non-Obstructive Hypoventilation, N(%) | < 1% | | P > 0.05 | No | No | |
| Never Smoker | 36.7 | 0.28 | P < 0.0001 | Yes | No | |
| Former Smoker | 43.0 | 0.09 | P < 0.0001 | No | No | -0.1203 |
| Current Smoker | 20.2 | -0.63 | P < 0.0001 | Yes | Yes | -0.6828 |

| | | | | | |
|---|---|---|---|---|---|
| Serotonin and Norepinephrine Reuptake Inhibitors, N(%) | 13.8 | -0.16 | P < 0.0001 | Yes | No |
| Spleen Removed, N(%) | < 1% | | P > 0.05 | No | No |
| Selective Serotonin Reuptake Inhibitors, N(%) | 20.1 | -0.09 | P < 0.0001 | No | No |
| Statin, N(%) | 48.4 | -0.09 | P < 0.0001 | Yes | No |
| Tested positive, N(%) | 19.6 | | P < 0.0001 | No | |
| Tocilizumab, N(%) | < 1% | | P > 0.05 | No | No |
| Tricyclic, N(%) | 4.7 | -0.17 | P < 0.0001 | Yes | No |
| Urinary Stones, N(%) | 9.7 | -0.13 | P < 0.0001 | Yes | No |
| Valsartan, N(%) | 1.5 | -0.01 | P > 0.05 | No | No |
| Vasopressin, N(%) | < 1% | | P > 0.05 | No | No |
| Vasopressor, N(%) | 2.1 | -0.15 | P < 0.0001 | Yes | No |
| Vecuronium, N(%) | < 1% | | P > 0.05 | No | No |
| Venous Thromboembolism, N(%) | 2.5 | -0.01 | P > 0.05 | No | No |

**Table S2b.** List of variables studied and results of feature selection (FS) from prevalence, Univariate Analysis, LASSO, and sequential forward stepwise selection for Unimputed data.

| Variables | Prevalence (%) | Univariate Analysis | | LASSO | Forward Selection | Logistic regression Coefficient |
|---|---|---|---|---|---|---|
| | | Beta | pValue | | | |
| Sex (Male) | 89.0 | | P < 0.0001 | Yes | No | |
| Age | | | | | | |
| Age 18–30 | 2.7 | | P < 0.0001 | Yes | No | |
| Age 30–40 | 9.6 | | P < 0.0001 | Yes | No | |
| Age 40–50 | 9.8 | | P < 0.0001 | Yes | No | |
| Age 50–65 | 28.6 | | P < 0.0001 | No | No | |
| Age 65–75 | 31.9 | | P < 0.0001 | Yes | No | |
| Age 75–85 | 12.9 | | P < 0.0001 | No | No | |
| Age > 85 | 4.5 | | P < 0.0001 | Yes | Yes | 0.2836 |
| Race | | | | | | |
| White | 68.3 | | P < 0.0001 | Yes | No | |
| Black | 22.5 | | P > 0.05 | No | No | |
| Other | 9.3 | | P < 0.0001 | No | No | |
| Ethnicity | | | | | | |
| Hispanic or Latino, N(%) | 8.4 | | P < 0.0001 | Yes | No | |
| BMI | | | | | | |
| BMI < 18.5 | 1.5 | | P < 0.0001 | Yes | Yes | -0.3154 |
| 18.5 ≤ BMI <30 | 52.7 | | P < 0.0001 | No | No | |
| BMI ≥ 30 | 45.8 | | P < 0.0001 | Yes | Yes | 0.2726 |
| Acetyl Cholinesterase (ACE) Inhibitor, N(%) | 29.2 | | P < 0.0001 | Yes | No | |
| Acetaminophen, N(%) | 40.1 | | P < 0.0001 | Yes | No | |
| Acute Cardiac Injury, N(%) | 2.4 | | P < 0.0001 | Yes | No | |
| Acute Liver Injury, N(%) | < 1% | | P > 0.05 | No | No | |
| Acute Myocardial Infarction, N(%) | 2.4 | | P < 0.0001 | No | No | |
| Acute Respiratory Failure, N(%) | 5.1 | | P > 0.05 | No | No | |
| Acute Rheumatic Heart Disease, N(%) | < 1% | | P > 0.05 | No | No | |
| Androgen deprivation therapy, N(%) | 1.2 | | P < 0.0001 | Yes | No | |
| Acute Kidney Failure, N(%) | 7.5 | | P = 0.091 | No | No | |
| Albumin, N(%) | < 1% | | P > 0.05 | No | No | |
| Alcohol Dependence, N(%) | 23.8 | | P < 0.0001 | No | No | |
| Allopurinol, N(%) | 6.3 | | P < 0.0001 | Yes | No | |
| Alphablocker, N(%) | 29.4 | | P < 0.0001 | No | No | |
| Antianginal, N(%) | 8.6 | | P < 0.0001 | No | No | |
| Antiarrhythmic, N(%) | 2.5 | | P < 0.0001 | No | No | |
| Antibiotic, N(%) | 43.8 | | P < 0.0001 | Yes | Yes | |

| | | | | | |
|---|---|---|---|---|---|
| Anticoagulant Inpatient, N(%) | 15.1 | P < 0.0001 | No | No | |
| Anticoagulant Outpatient, N(%) | 16.5 | P < 0.0001 | Yes | No | |
| Antihistamine, N(%) | 43.2 | P < 0.0001 | No | No | |
| Antipsychotic, N(%) | 10.1 | P < 0.0001 | Yes | No | |
| Antiviral, N(%) | 8.6 | P < 0.0001 | No | No | |
| Anxiety, N(%) | 23.5 | P < 0.0001 | No | No | |
| Angiotensin II Receptor Blocker, N(%) | 13.7 | P < 0.0001 | No | No | |
| Acute Respiratory Distress Syndrome, N(%) | < 1% | P > 0.05 | No | No | |
| Aromatase Inhibitors, N(%) | < 1% | P > 0.05 | No | No | |
| Asthma, N(%) | 7.2 | P > 0.05 | No | No | |
| Azithromycin, N(%) | 10.8 | P < 0.0001 | Yes | No | |
| Bacillus Calmette-Guerin, N(%) | < 1% | P > 0.05 | No | No | |
| Benzodiazepine, N(%) | 13.1 | P < 0.0001 | Yes | No | |
| Beta Agonist Nebulizer, N(%) | 10.2 | P < 0.0001 | Yes | No | |
| Beta Blocker, N(%) | 34.1 | P < 0.0001 | Yes | No | |
| Bipolar Disorder, N(%) | 4.8 | P < 0.0001 | Yes | No | |
| Bronchitis, N(%) | 7.7 | P < 0.0001 | Yes | No | |
| Bronchodilator, N(%) | 26.4 | P < 0.0001 | No | No | |
| Coronary Atherosclerosis and other Heart Disease, N(%) | 21.2 | P < 0.0001 | Yes | No | |
| Cancer, N(%) | 22.5 | P < 0.0001 | Yes | Yes | -0.3232 |
| Cardiomyopathy, N(%) | 3.8 | P < 0.0001 | Yes | No | |
| Calcium Channel Blocker, N(%) | 26.8 | P < 0.0001 | No | No | |
| Cerebrovascular Disease, N(%) | 2.2 | P = 0.0006 | No | No | |
| Congestive Heart Failure, N(%) | 8.3 | P < 0.0001 | Yes | No | |
| Chloroquine, N(%) | < 1% | P > 0.05 | No | No | |
| Chronic hepatitis, N(%) | < 1% | P > 0.05 | No | No | |
| Chronic Lung Disease, N(%) | 34.5 | P < 0.0001 | Yes | No | |
| Chronic Neurological Disease, N(%) | 3.6 | P < 0.0001 | Yes | No | |
| Chronic Rheumatic Heart Disease, N(%) | 1.0 | P > 0.05 | Yes | No | |
| Cirrhosis, N(%) | 2.7 | P < 0.0001 | Yes | No | |
| Cis-atracurium, N(%) | < 1% | P > 0.05 | No | No | |
| Chronic Kidney Disease, N(%) | 13.9 | P < 0.0001 | Yes | No | |
| Chronic Kidney Failure, N(%) | 1.6 | P > 0.05 | No | No | |
| Colchicine, N(%) | 2.6 | P > 0.05 | No | No | |
| Complex Sleep Apnea, N(%) | < 1% | P > 0.05 | No | No | |
| Chronic Obstructive Pulmonary Disease, N(%) | 19.1 | P < 0.0001 | No | No | |
| Corticosteroid, N(%) | 39.2 | P < 0.0001 | Yes | No | |
| Central sleep Apnea Primary, N(%) | < 1% | P > 0.05 | No | No | |
| Central serous retinopathy, N(%) | < 1% | P > 0.05 | No | No | |
| Cardiovascular Disease, N(%) | 35.7 | P < 0.0001 | Yes | No | |

| | | | | | |
|---|---|---|---|---|---|
| Degarelix, N(%) | < 1% | P > 0.05 | No | No | |
| Dementia, N(%) | 4.2 | P < 0.0001 | Yes | Yes | 0.3496 |
| Dexamethasone, N(%) | 3.9 | P < 0.0001 | No | No | |
| Diabetes Any, N(%) | 33.2 | P < 0.0001 | Yes | No | |
| Diabetes Other, N(%) | 3.3 | P = 0.0006 | Yes | No | |
| Diabetes Type 1, N(%) | 1.9 | P > 0.05 | No | No | |
| Diabetes Type 2, N(%) | 33.0 | P < 0.0001 | Yes | No | |
| Diabetes With Complications, N(%) | 23.5 | P < 0.0001 | No | No | |
| Diabetes Without Complications, N(%) | 30.0 | P < 0.0001 | No | No | |
| Dobutamine, N(%) | < 1% | P > 0.05 | No | No | |
| Non-Alcohol Drug Dependence, N(%) | 6.5 | P < 0.0001 | Yes | No | |
| Eculizumab, N(%) | < 1% | P > 0.05 | No | No | |
| Emphysema, N(%) | 2.3 | P < 0.0001 | No | No | |
| Epinephrine, N(%) | 2.8 | P < 0.0001 | Yes | No | |
| Estrogen, N(%) | 2.2 | P < 0.0001 | No | No | |
| Famotidine, N(%) | 8.9 | P < 0.0001 | No | No | |
| Furosemide, N(%) | 12.1 | P < 0.0001 | No | No | |
| Guillain-Barre Syndrome, N(%) | < 1% | P > 0.05 | No | No | |
| Glucocorticoid, N(%) | 23.9 | P < 0.0001 | No | No | |
| Histamine 2 Receptor Blocker, N(%) | 9.2 | P < 0.0001 | Yes | No | |
| Hydrochlorothiazide, N(%) | 16.3 | P = 0.0016 | No | No | |
| Heart Disease, N(%) | 26.4 | P < 0.0001 | Yes | No | |
| Heart Failure, N(%) | 10.3 | P < 0.0001 | No | No | |
| Hemorrhagic Stroke, N(%) | < 1% | P > 0.05 | No | No | |
| Human Immunodeficiency Virus, N(%) | < 1% | P > 0.05 | No | No | |
| Hypertension, N(%) | 63.9 | P < 0.0001 | Yes | No | |
| Hydroxychloroquine, N(%) | < 1% | P > 0.05 | No | No | |
| Hyperlipidemia, N(%) | 60.3 | P < 0.0001 | Yes | No | |
| Inflammatory bowel Disease, N(%) | 3.6 | P < 0.0001 | Yes | No | |
| Inhaled Corticosteroids, N(%) | 2.2 | P < 0.0001 | Yes | No | |
| Inhaled Corticosteroids and Long-Acting Beta-Agonist, N(%) | 11.7 | P < 0.0001 | Yes | No | |
| Induction Ventilation Medications, N(%) | 2.0 | P < 0.0001 | No | No | |
| Influenza, N(%) | 2.0 | P = 0.00012 | Yes | No | |
| Inhaled Budesonide, N(%) | < 1% | P > 0.05 | No | No | |
| Inhaled Epo-prostenol, N(%) | < 1% | P > 0.05 | No | No | |
| Ischemic Stroke, N(%) | 4.9 | P =0.0003 | Yes | No | |
| Isoproterenol, N(%) | < 1% | P > 0.05 | No | No | |
| Kidney Disease All, N(%) | 23.3 | P < 0.0001 | Yes | No | |
| Long-Acting Beta-Agonist, N(%) | < 1% | P > 0.05 | No | No | |
| Liver Disease, N(%) | 7.7 | P < 0.0001 | Yes | No | |

| | | | | | |
|---|---|---|---|---|---|
| Lopinavir, N(%) | < 1% | P > 0.05 | No | No | |
| Losartan, N(%) | 12.4 | P < 0.0001 | No | No | |
| Lower Respiratory Infection, N(%) | 8.4 | P < 0.0001 | Yes | Yes | 0.0907 |
| Major Depressive Disorder, N(%) | 37.1 | P < 0.0001 | Yes | No | |
| Measles, N(%) | < 1% | P > 0.05 | No | No | |
| Metformin, N(%) | 20.0 | P < 0.0001 | Yes | No | |
| Milrinone, N(%) | < 1% | P > 0.05 | No | No | |
| Multiple Sclerosis, N(%) | < 1% | P > 0.05 | No | No | |
| Nephrosis, N(%) | 3.0 | P > 0.05 | No | No | |
| Norepinephrine, N(%) | < 1% | P > 0.05 | No | No | |
| Non-steroidal Anti-Inflammatory Drug, N(%) | 63.0 | P < 0.0001 | Yes | No | |
| Obesity Hypoventilation, N(%) | < 1% | P > 0.05 | No | No | |
| Opioid, N(%) | 6.0 | P < 0.0001 | Yes | No | |
| Obstructive Sleep Apnea, N(%) | 31.8 | P < 0.0001 | No | No | |
| Other and Ill-Defined Heart Disease, N(%) | 3.1 | P < 0.0001 | No | No | |
| Overweight, N(%) | 6.8 | P < 0.0001 | Yes | No | |
| Peripheral Artery Disease, N(%) | 12.7 | P < 0.0001 | Yes | No | |
| Platelet Aggregation Inhibitor, N(%) | 7.2 | P < 0.0001 | Yes | No | |
| Parkinsons Disease, N(%) | 1.3 | P < 0.0001 | Yes | No | |
| Polycystic Ovary Syndrome, N(%) | < 1% | P > 0.05 | No | No | |
| Phenylephrine, N(%) | 1.9 | P < 0.0001 | Yes | Yes | -0.2779 |
| Pneumonia, N(%) | 5.7 | P < 0.0001 | Yes | Yes | 0.1789 |
| Ventilator-Associated Pneumonia, N(%) | < 1% | P > 0.05 | No | No | |
| Proton Pump Inhibitor, N(%) | 38.9 | P < 0.0001 | Yes | No | |
| Pregnancy, N(%) | 0.4 | P > 0.05 | No | No | |
| Pregnancy at Index, N(%) | < 1% | P > 0.05 | No | No | |
| Posttraumatic Stress Disorder, N(%) | 26.9 | P < 0.0001 | No | No | |
| Pulmonary Heart Disease, N(%) | 2.2 | P < 0.0001 | Yes | No | |
| Remdesivir, N(%) | < 1% | P > 0.05 | No | No | |
| Ribavirin, N(%) | < 1% | P > 0.05 | No | No | |
| Rosiglitazone, N(%) | < 1% | P > 0.05 | No | No | |
| Sarilumab, N(%) | < 1% | P > 0.05 | No | No | |
| Schizophrenia, N(%) | 2.8 | P < 0.0001 | Yes | No | |
| Septic Shock, N(%) | 2.6 | P < 0.0001 | Yes | Yes | 0.0652 |
| Selective Estrogen Receptor Degrader, N(%) | < 1% | P > 0.05 | No | No | |
| Selective Estrogen Receptor Modulators, N(%) | < 1% | P > 0.05 | No | No | |
| Sickle Cell Disease, N(%) | < 1% | P > 0.05 | No | No | |
| Sleep Related Non-Obstructive Hypoventilation, N(%) | < 1% | P > 0.05 | No | No | |
| Never Smoker | 36.0 | P < 0.0001 | Yes | No | |
| Former Smoker | 43.1 | P < 0.0001 | No | No | -0.1098 |
| Current Smoker | 20.9 | P < 0.0001 | Yes | Yes | -0.6943 |

| | | | | |
|---|---|---|---|---|
| Serotonin and Norepinephrine Reuptake Inhibitors, N(%) | 14.9 | P < 0.0001 | Yes | No |
| Spleen Removed, N(%) | < 1% | P > 0.05 | No | No |
| Selective Serotonin Reuptake Inhibitors, N(%) | 21.5 | P < 0.0001 | No | No |
| Statin, N(%) | 52.3 | P < 0.0001 | Yes | No |
| Tested positive, N(%) | 19.5 | P < 0.0001 | No | No |
| Tocilizumab, N(%) | < 1% | P > 0.05 | No | No |
| Tricyclic, N(%) | 5.1 | P < 0.0001 | Yes | No |
| Urinary Stones, N(%) | 10.4 | P < 0.0001 | Yes | No |
| Valsartan, N(%) | 1.6 | P > 0.05 | No | No |
| Vasopressin, N(%) | < 1% | P > 0.05 | No | No |
| Vasopressor, N(%) | 2.2 | P < 0.0001 | Yes | No |
| Vecuronium, N(%) | < 1% | P > 0.05 | No | No |
| Venous Thromboembolism, N(%) | 2.7 | P = 0.046 | Yes | No |

**Table S3.** Prevalence of patient characteristics by testing status

| Variables | Tested Negative n= 946,056 (80.4%) | Tested Positive n= 230,250 (19.6%) |
|---|---|---|
| Sex (Male) | 816322. (86.3) | 201077. (87.3) |
| Race | | |
| White | 620336. (65.6) | 150134. (65.2) |
| Black | 205536. (21.7) | 49658. (21.6) |
| Others | 120184. (12.7) | 30458. (13.2) |
| Ethnicity | | |
| Not Hispanic Or Latino | 806525. (85.3) | 192428. (83.6) |
| Hispanic Or Latino | 74221. (7.8) | 22124. (9.6) |
| Unknown | 65310. (6.9) | 15698. (6.8) |
| Age [Mean, SD] | | |
| Age 18–30 | 41230. (4.4) | 11148. (4.8) |
| Age 30–40 | 102685. (10.9) | 27340. (11.9) |
| Age 40–50 | 101603. (10.7) | 28539. (12.4) |
| Age 50–65 | 277380. (29.3) | 63293. (27.5) |
| Age 65–75 | 291438. (30.8) | 63142. (27.4) |
| Age 75–85 | 97509. (10.3) | 25179. (10.9) |
| Age > 85 | 34210. (3.6) | 11609. (5.0) |
| BMI [Mean, SD] | | |
| BMI < 18.5 | 15266. (1.6) | 2253. (1.0) |
| 18.5 ≤ BMI <30 | 517919. (54.7) | 110634. (48.0) |
| BMI ≥ 30 | 412871. (43.6) | 117363. (51.0) |
| **Pre-existing Conditions** | | |
| Acute Cardiac Injury, N(%) | 21745. (2.3) | 5003. (2.2) |
| Acute Liver Injury, N(%) | 1950. (0.2) | 343. (0.1) |
| Acute Myocardial Infarction, N(%) | 21699. (2.3) | 4993. (2.2) |
| Acute Respiratory Failure, N(%) | 45619. (4.8) | 11443. (5.0) |
| Acute Rheumatic Heart Disease, N(%) | 53. (0.0) | 9. (0.0) |
| Acute Kidney Failure, N(%) | 67036. (7.1) | 16318. (7.1) |
| Alcohol Dependence, N(%) | 214249. (22.6) | 45535. (19.8) |
| Anxiety, N(%) | 210951. (22.3) | 47013. (20.4) |
| Acute Respiratory Distress Syndrome, N(%) | 552. (0.1) | 248. (0.1) |
| Asthma, N(%) | 63349. (6.7) | 15274. (6.6) |
| Bipolar Disorder, N(%) | 45105. (4.8) | 7957. (3.5) |
| Breastfeeding, N(%) | 670. (0.1) | 209. (0.1) |
| Breastfeed At Index | 14. (0.0) | 4. (0.0) |
| Bronchitis, N(%) | 68086. (7.2) | 15691. (6.8) |
| Coronary Atherosclerosis and other Heart Disease, N(%) | 187437. (19.8) | 43765. (19.0) |
| Cancer, N(%) | 207470. (21.9) | 39182. (17.0) |
| Cardiomyopathy, N(%) | 34151. (3.6) | 6968. (3.0) |

| | | |
|---|---|---|
| Cerebrovascular Disease, N(%) | 19361. (2.0) | 4532. (2.0) |
| Congestive Heart Failure, N(%) | 74701. (7.9) | 16781. (7.3) |
| Chronic hepatitis, N(%) | 3201. (0.3) | 534. (0.2) |
| Chronic Lung Disease, N(%) | 309740. (32.7) | 66367. (28.8) |
| Chronic Neurological Disease, N(%) | 32078. (3.4) | 8296. (3.6) |
| Chronic Rheumatic Heart Disease, N(%) | 9166. (1.0) | 1912. (0.8) |
| Cirrhosis, N(%) | 26015. (2.7) | 4290. (1.9) |
| Chronic Kidney Disease, N(%) | 122425. (12.9) | 30822. (13.4) |
| Chronic Kidney Failure, N(%) | 14384. (1.5) | 3611. (1.6) |
| Complex Sleep Apnea, N(%) | 162. (0.0) | 29. (0.0) |
| Chronic Obstructive Pulmonary Disease, N(%) | 173612. (18.4) | 34497. (15.0) |
| Central sleep Apnea Primary, N(%) | 3440. (0.4) | 760. (0.3) |
| Central serous retinopathy, N(%) | 2141. (0.2) | 416. (0.2) |
| Cardiovascular Disease, N(%) | 319657. (33.8) | 71859. (31.2) |
| Dementia, N(%) | 36320. (3.8) | 12739. (5.5) |
| Diabetes Any, N(%) | 288130. (30.5) | 76563. (33.3) |
| Diabetes Other, N(%) | 29557. (3.1) | 6918. (3.0) |
| Diabetes Type 1, N(%) | 16385. (1.7) | 4000. (1.7) |
| Diabetes Type 2, N(%) | 285834. (30.2) | 76017. (33.0) |
| Diabetes With Complications, N(%) | 203835. (21.5) | 53451. (23.2) |
| Diabetes Without Complications, N(%) | 259149. (27.4) | 68705. (29.8) |
| Drug Dependence, N(%) | 62290. (6.6) | 9129. (4.0) |
| Emphysema, N(%) | 20877. (2.2) | 3698. (1.6) |
| Guillain-Barre Syndrome, N(%) | 553. (0.1) | 115. (0.0) |
| Heart Disease, N(%) | 234426. (24.8) | 54563. (23.7) |
| Heart Failure, N(%) | 92456. (9.8) | 21187. (9.2) |
| Hemorrhagic Stroke, N(%) | 2395. (0.3) | 610. (0.3) |
| Human Immunodeficiency Virus, N(%) | 8895. (0.9) | 1789. (0.8) |
| Hypertension, N(%) | 562605. (59.5) | 136155. (59.1) |
| Hyperlipidemia, N(%) | 524911. (55.5) | 130877. (56.8) |
| Inflammatory bowel Disease, N(%) | 32661. (3.5) | 6698. (2.9) |
| Influenza, N(%) | 17326. (1.8) | 4564. (2.0) |
| Ischemic Stroke, N(%) | 44261. (4.7) | 10599. (4.6) |
| Kidney Disease All, N(%) | 207173. (21.9) | 49216. (21.4) |
| Liver Disease, N(%) | 69995. (7.4) | 14427. (6.3) |
| Lower Respiratory Infection, N(%) | 73546. (7.8) | 19666. (8.5) |
| Major Depressive Disorder, N(%) | 333472. (35.2) | 73817. (32.1) |
| Measles, N(%) | 19. (0.0) | 7. (0.0) |
| Multiple Sclerosis, N(%) | 4108. (0.4) | 901. (0.4) |
| Nephrosis, N(%) | 26402. (2.8) | 6359. (2.8) |
| Obesity Hypoventilation, N(%) | 4005. (0.4) | 1082. (0.5) |
| Obstructive Sleep Apnea, N(%) | 276999. (29.3) | 70007. (30.4) |

| | | |
|---|---|---|
| Other and Ill-Defined Heart Disease, N(%) | 28123. (3.0) | 6277. (2.7) |
| Overweight, N(%) | 58473. (6.2) | 18480. (8.0) |
| Peripheral Artery Disease, N(%) | 115486. (12.2) | 23607. (10.3) |
| Parkinsons Disease, N(%) | 11431. (1.2) | 3182. (1.4) |
| Polycystic Ovary Syndrome, N(%) | 1488. (0.2) | 364. (0.2) |
| Pneumonia, N(%) | 49597. (5.2) | 13977. (6.1) |
| Ventilator-Associated Pneumonia, N(%) | 234. (0.0) | 53. (0.0) |
| Pregnancy, N(%) | 3403. (0.4) | 1020. (0.4) |
| Pregnancy at Index, N(%) | 895. (0.1) | 299. (0.1) |
| Posttraumatic Stress Disorder, N(%) | 241243. (25.5) | 54029. (23.5) |
| Pulmonary Heart Disease, N(%) | 20401. (2.2) | 4294. (1.9) |
| Schizophrenia, N(%) | 26709. (2.8) | 5153. (2.2) |
| Septic Shock, N(%) | 23110. (2.4) | 6304. (2.7) |
| Sickle Cell Disease, N(%) | 1562. (0.2) | 354. (0.2) |
| Sleep Related Non-Obstructive Hypoventilation, N(%) | 3895. (0.4) | 868. (0.4) |
| Spleen Removed, N(%) | 1451. (0.2) | 273. (0.1) |
| Urinary Stones, N(%) | 94118. (9.9) | 20188. (8.8) |
| Venous Thromboembolism, N(%) | 23786. (2.5) | 5649. (2.5) |
| **Pre-infection Medications** | | |
| Acetyl Cholinesterase (ACE) Inhibitor, N(%) | 258742. (27.3) | 60410. (26.2) |
| Acetaminophen, N(%) | 366495. (38.7) | 77203. (33.5) |
| Androgen deprivation therapy, N(%) | 10769. (1.1) | 1987. (0.9) |
| Albumin, N(%) | 6353. (0.7) | 1270. (0.6) |
| Allopurinol, N(%) | 54674. (5.8) | 14472. (6.3) |
| Alphablocker, N(%) | 262869. (27.8) | 58258. (25.3) |
| Antianginal, N(%) | 76874. (8.1) | 16990. (7.4) |
| Antiarrhythmic, N(%) | 22298. (2.4) | 4672. (2.0) |
| Antibiotic, N(%) | 399342. (42.2) | 84288. (36.6) |
| Anticoagulant Inpatient, N(%) | 139376. (14.7) | 28287. (12.3) |
| Anticoagulant Outpatient, N(%) | 147393. (15.6) | 33077. (14.4) |
| Antihistamine, N(%) | 387554. (41.0) | 86167. (37.4) |
| Antipsychotic, N(%) | 93437. (9.9) | 17992. (7.8) |
| Antiviral, N(%) | 78971. (8.3) | 18154. (7.9) |
| Angiotensin II Receptor Blocker, N(%) | 119267. (12.6) | 30096. (13.1) |
| Aromatase Inhibitors, N(%) | 1401. (0.1) | 253. (0.1) |
| Azithromycin, N(%) | 96217. (10.2) | 22343. (9.7) |
| Bacillus Calmette-Guerin, N(%) | 1864. (0.2) | 214. (0.1) |
| Benzodiazepine, N(%) | 120923. (12.8) | 22619. (9.8) |
| Beta Agonist Nebulizer, N(%) | 93498. (9.9) | 19198. (8.3) |
| Beta Blocker, N(%) | 305095. (32.2) | 68591. (29.8) |
| Bronchodilator, N(%) | 237864. (25.1) | 50376. (21.9) |
| Calcium Channel Blocker, N(%) | 238232. (25.2) | 54619. (23.7) |

| | | |
|---|---|---|
| Chloroquine, N(%) | 15. (0.0) | 5. (0.0) |
| Cis-atracurium, N(%) | 220. (0.0) | 31. (0.0) |
| Colchicine, N(%) | 23016. (2.4) | 5520. (2.4) |
| Corticosteroid, N(%) | 351344. (37.1) | 77266. (33.6) |
| Degarelix, N(%)yrs | 180. (0.0) | 29. (0.0) |
| Dexamethasone, N(%) | 36265. (3.8) | 7194. (3.1) |
| Dobutamine, N(%) | 1096. (0.1) | 210. (0.1) |
| Eculizumab, N(%) | 15. (0.0) | 3. (0.0) |
| Epinephrine, N(%) | 25280. (2.7) | 5415. (2.4) |
| Estrogen, N(%) | 19990. (2.1) | 4406. (1.9) |
| Famotidine, N(%) | 80219. (8.5) | 17206. (7.5) |
| Furosemide, N(%) | 108865. (11.5) | 24734. (10.7) |
| Glucocorticoid, N(%) | 216312. (22.9) | 46602. (20.2) |
| Histamine 2 Receptor Blocker, N(%) | 83648. (8.8) | 16959. (7.4) |
| Hydrochlorothiazide, N(%) | 143246. (15.1) | 34283. (14.9) |
| Hydroxychloroquine, N(%) | 7416. (0.8) | 1510. (0.7) |
| Inhaled Corticosteroids, N(%) | 19658. (2.1) | 4182. (1.8) |
| Inhaled Corticosteroids and Long-Acting Beta-Agonist, N(%) | 104819. (11.1) | 22246. (9.7) |
| Induction Ventilation Medications, N(%) | 19185. (2.0) | 3432. (1.5) |
| Inhaled Budesonide, N(%) | 3306. (0.3) | 714. (0.3) |
| Inhaled Epo-prostenol, N(%) | 94. (0.0) | 26. (0.0) |
| Isoproterenol, N(%) | 112. (0.0) | 20. (0.0) |
| Long-Acting Beta-Agonist, N(%) | 3562. (0.4) | 686. (0.3) |
| Lopinavir, N(%) | 69. (0.0) | 10. (0.0) |
| Losartan, N(%) | 107423. (11.4) | 27223. (11.8) |
| Metformin, N(%) | 171484. (18.1) | 45995. (20.0) |
| Milrinone, N(%) | 556. (0.1) | 115. (0.0) |
| Norepinephrine, N(%) | 4509. (0.5) | 982. (0.4) |
| Non-steroidal Anti-Inflammatory Drug, N(%) | 565074. (59.7) | 128784. (55.9) |
| Opioid, N(%) | 56275. (5.9) | 9747. (4.2) |
| Platelet Aggregation Inhibitor, N(%) | 64141. (6.8) | 14702. (6.4) |
| Phenylephrine, N(%) | 17529. (1.9) | 3220. (1.4) |
| Proton Pump Inhibitor, N(%) | 348099. (36.8) | 76583. (33.3) |
| Remdesivir, N(%) | 8. (0.0) | 40. (0.0) |
| Ribavirin, N(%) | 136. (0.0) | 13. (0.0) |
| Rosiglitazone, N(%) | 0. (0.0) | 0. (0.0) |
| Sarilumab, N(%) | 11. (0.0) | 1. (0.0) |
| Selective Estrogen Receptor Degrader, N(%) | 46. (0.0) | 4. (0.0) |
| Selective Estrogen Receptor Modulators, N(%) | 804. (0.1) | 157. (0.1) |
| Serotonin and Norepinephrine Reuptake Inhibitors, N(%) | 134438. (14.2) | 28062. (12.2) |
| Selective Serotonin Reuptake Inhibitors, N(%) | 192925. (20.4) | 43320. (18.8) |

| | | |
|---|---|---|
| Statin, N(%) | 461512. (48.8) | 108101. (46.9) |
| Tocilizumab, N(%) | 342. (0.0) | 84. (0.0) |
| Tricyclic, N(%) | 45863. (4.8) | 9404. (4.1) |
| Valsartan, N(%) | 14252. (1.5) | 3443. (1.5) |
| Vasopressin, N(%) | 1582. (0.2) | 328. (0.1) |
| Vasopressor, N(%) | 20231. (2.1) | 4269. (1.9) |
| Vecuronium, N(%) | 60. (0.0) | 7. (0.0) |
| Never Smokers | 334659. (35.4) | 97419. (42.3) |
| Former Smokers | 402724. (42.6) | 103482. (44.9) |
| Current Smokers | 208673. (22.1) | 29349. (12.7) |

**Table S4:** Pre-existing respiratory diseases

| Variables | Never Smoker n = 423972 | Former Smoker n =505465 | p-Value | Current Smoker n=245127 | p-Value | Unknown Smoker n=130427 | p-Value |
|---|---|---|---|---|---|---|---|
| Chronic Lung Disease, N(%) | 110297 (26.0) | 187984 (37.2) | p<0.0001 | 101492 (41.4) | p<0.0001 | 12233 (9.4) | p<0.0001 |
| Obstructive Sleep Apnea, N(%) | 143519 (33.9) | 169011 (33.4) | p<0.0001 | 61377 (25) | p<0.0001 | 11160 (8.6) | p<0.0001 |
| Chronic Obstructive Pulmonary Disease, N(%) | 39445 (9.3) | 111223 (22) | p<0.0001 | 69962 (28.5) | p<0.0001 | 6569 (5) | p<0.0001 |
| Asthma, N(%) | 36058 (8.5) | 35361 (7) | p<0.0001 | 12304 (5) | p<0.0001 | 2855 (2.2) | p<0.0001 |
| Lower Respiratory Infection, N(%) | 35529 (8.4) | 41361 (8.2) | p<0.0001 | 19316 (7.9) | p<0.0001 | 4587 (3.5) | p<0.0001 |
| Bronchitis, N(%) | 27671 (6.5) | 39182 (7.8) | p<0.0001 | 20514 (8.4) | p<0.0001 | 2936 (2.3) | p<0.0001 |
| Pneumonia, N(%) | 18381 (4.3) | 35078 (6.9) | p<0.0001 | 15428 (6.3) | p<0.0001 | 3522 (2.7) | p<0.0001 |
| Influenza, N(%) | 7785 (1.8) | 9463 (1.9) | p<0.0001 | 4980 (2) | p<0.0001 | 1263 (1) | p<0.0001 |
| Pulmonary Heart Disease, N(%) | 7235 (1.7) | 13973 (2.8) | p<0.0001 | 4325 (1.8) | p = 0.003 | 1012 (0.8) | p<0.0001 |
| Acute Respiratory Failure, N(%) | 7207 (1.7) | 16829 (3.3) | p<0.0001 | 8193 (3.3) | p<0.0001 | 1721 (1.3) | p<0.0001 |
| Emphysema, N(%) | 3374 (0.8) | 13553 (2.7) | p<0.0001 | 8966 (3.7) | p<0.0001 | 647 (0.5) | p<0.0001 |
| Acute Respiratory Distress Syndrome, N(%) | 201 (0) | 378 (0.1) | | 175 (0.1) | | 35 (0) | |
| Complex Sleep Apnea, N(%) | 82 (0) | 90 (0) | | 33 (0) | | 7 (0) | |
| Ventilator-Associated Pneumonia, N(%) | 61 (0) | 136 (0) | | 64 (0) | | 30 (0) | |

**Table S5.** Association between smoking status and COVID-19 positivity stratify by age for imputed and unimputed data

| Variables | Odds Ratio (95%Confidence intervals) | |
|---|---|---|
| | Unadjusted | Adjusted |
| **Imputed Unknown Smoker to Never, Former, and Current Smoker** | | |
| Model 1† | | |
| Former Smoker vs. Never Smoker | **0.88 (0.87, 0.89)** | **0.89 (0.88, 0.90)** |
| Current Smoker vs. Never Smoker | **0.48 (0.47,0.49)** | **0.51 (0.50,0.52)** |
| Model 2‡ | | |
| *Age ≥ 65* | | |
| Former Smoker vs. Never Smoker | 0.99 (0.97, 1.00) | 0.98 (0.97, 1.00) |
| Current Smoker vs. Never Smoker | **0.50 (0.49, 0.51)** | **0.52 (0.51, 0.53)** |
| *Age < 65* | | |
| Former Smoker vs. Never Smoker | **0.83 (0.81, 0.84)** | **0.83 (0.81, 0.84)** |
| Current Smoker vs. Never Smoker | **0.47 (0.46, 0.48)** | **0.48 (0.47, 0.49)** |
| **Unimputed Unknown Smoker** | | |
| Model 1 † | | |
| Former Smoker vs. Never Smoker | **0.89 (0.88, 090)** | **0.90 (0.89, 0.91)** |
| Current Smoker vs. Never Smoker | **0.47 (0.46, 0.48)** | **0.50 (0.49, 0.51)** |
| Unknown Smoker vs. Never Smoker | **0.89 (0.88, 0.90)** | **0.93 (0.91, 0.94)** |
| Model 2‡ | | |
| *Age ≥ 65* | | |
| Former Smoker vs. Never Smoker | 1.01 (0.99, 1.02) | 1.00 (0.99, 1.01) |
| Current Smoker vs. Never Smoker | **0.50 (0.49, 0.51)** | **0.51 (0.50, 0.53)** |
| Unknown Smoker vs. Never Smoker | **1.27 (1.24, 1.31)** | **1.22 (1.18, 1.25)** |
| *Age < 65* | | |
| Former Smoker vs. Never Smoker | **0.83 (0.82, 0.84)** | **0.83 (0.82, 0.84)** |
| Current Smoker vs. Never Smoker | **0.45 (0.44, 0.46)** | **0.48 (0.47, 0.49)** |
| Unknown Smoker vs. Never Smoker | **0.75 (0.74, 0.76)** | **0.82 (0.80, 0.83)** |

† Model 1 adjusted by Age ≥ 85, BMI < 18.5, BMI ≥ 30, Sex, Former Smoker, Current Smoker, Cancer, Dementia, Hispanic or Latino, Low Respiratory Infections, Pneumonia, Phenylephrine, and Septic Shock.

‡ Model 2 stratified by age and adjusted by BMI < 18.5, BMI ≥ 30, Sex, Former Smoker, Current Smoker, Cancer, Dementia, Hispanic or Latino, Low Respiratory Infections, Pneumonia, Phenylephrine, and Septic Shock.

Bold indicate reduced risk of SARS-CoV-2 infection.
